# Methyl 1*H*-1,2,3-triazole-4-carboxyl­ate

**DOI:** 10.1107/S1600536809000877

**Published:** 2009-01-14

**Authors:** K. Prabakaran, T. Maiyalagan, Venkatesha R. Hathwar, Canan Kazak, F. Nawaz Khan

**Affiliations:** a Chemistry Division, School of Science and Humanities, VIT University, Vellore 632 014, Tamil Nadu, India; b Solid State and Structural Chemistry Unit, Indian Institute of Science, Bangalore 560 012, Karnataka, India; c Ondokuz Mayıs University, Arts and Sciences Faculty, Department of Physics, 55139 Samsun, Turkey

## Abstract

The title compound, C_4_H_5_N_3_O_2_, features an essentially planar mol­ecule (r.m.s. deviation for all non-H atoms = 0.013 Å). The crystal structure is stabilized by inter­molecular N—H⋯O hydrogen bonds and π–π stacking inter­actions (centroid–centroid distance 3.882 Å).

## Related literature

For general background, see: Abu-Orabi *et al.* (1989 ▶); Fan & Katritzky (1996 ▶); Dehne (1994 ▶). For a related structure, see: Wang *et al.* (1998 ▶).
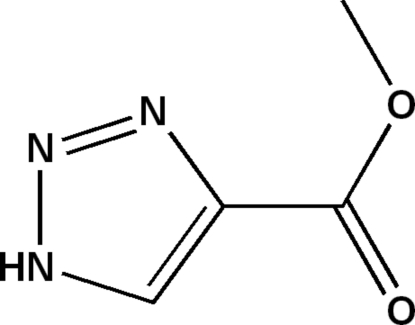


## Experimental

### 

#### Crystal data

C_4_H_5_N_3_O_2_
*M* *_r_* = 127.11
Monoclinic, 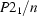

*a* = 3.8823 (7) Å
*b* = 17.499 (3) Å
*c* = 8.8171 (17) Å
β = 100.938 (3)°
*V* = 588.12 (19) Å^3^
*Z* = 4
Mo *K*α radiation
μ = 0.12 mm^−1^
*T* = 290 (2) K
0.30 × 0.23 × 0.20 mm

#### Data collection

Bruker SMART CCD area-detector diffractometer
Absorption correction: multi-scan (*SADABS*; Bruker, 2004 ▶) *T*_min_ = 0.956, *T*_max_ = 0.977
4285 measured reflections
1098 independent reflections
917 reflections with *I* > 2σ(*I*)
*R*_int_ = 0.016

#### Refinement

*R*[*F* ^2^ > 2σ(*F* ^2^)] = 0.039
*wR*(*F* ^2^) = 0.116
*S* = 1.06
1098 reflections
91 parameters
H atoms treated by a mixture of independent and constrained refinement
Δρ_max_ = 0.20 e Å^−3^
Δρ_min_ = −0.12 e Å^−3^

### 

Data collection: *SMART* (Bruker, 2004 ▶); cell refinement: *SAINT* (Bruker, 2004 ▶); data reduction: *SAINT*; program(s) used to solve structure: *SHELXS97* (Sheldrick, 2008 ▶); program(s) used to refine structure: *SHELXL97* (Sheldrick, 2008 ▶); molecular graphics: *ORTEP-3* (Farrugia, 1999 ▶) and *CAMERON* (Watkin *et al.*, 1993 ▶); software used to prepare material for publication: *PLATON* (Spek, 2003 ▶).

## Supplementary Material

Crystal structure: contains datablocks global, I. DOI: 10.1107/S1600536809000877/bt2847sup1.cif

Structure factors: contains datablocks I. DOI: 10.1107/S1600536809000877/bt2847Isup2.hkl

Additional supplementary materials:  crystallographic information; 3D view; checkCIF report

## Figures and Tables

**Table 1:** Hydrogen-bond geometry (Å, °)

*D*—H⋯*A*	*D*—H	H⋯*A*	*D*⋯*A*	*D*—H⋯*A*
N3—H3*N*⋯O1^i^	0.896 (19)	1.980 (19)	2.8659 (19)	169.56 (18)

Symmetry code: (i) 
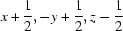
.

**Fig. 1.**
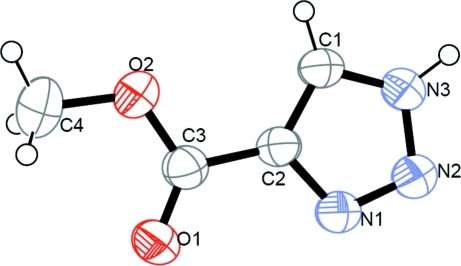
ORTEP diagram of the asymmetric unit of the title compound with 50% probability displacement ellipsoids.

**Fig. 2.**
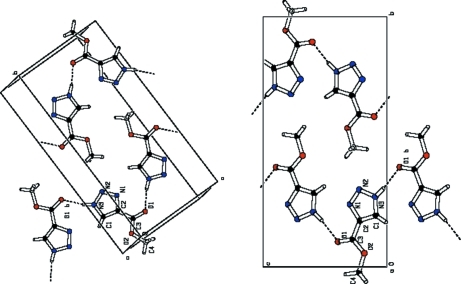
The crystal packing diagram of the title compound. The dotted lines indicate intermolecular N—H···O hydrogen bonds.

## supplementary crystallographic information

### Comment

Triazoles play an important role in pharmaceuticals, agrochemicals, dyes, photographic materials, and in corrosion inhibition (Fan & Katritzky, 1996; Dehne, 1994; Abu-Orabi *et al.*, 1989).

The crystal structure of the title compound is stabilized by intermolecular N—H···O  hydrogen bonds and   π^···^π stacking interactions between the triazole rings at (symmetry operator 1+*X*,Y,*Z*; centroid-centroid distance 3.882 Å).

### Experimental

A mixture of methylpropiolate, 1 and trimethylsilylazide, 2 were heated at 100 °C till the completion of reaction, monitored by TLC. Then reaction mixture was cooled and methanol was added dropwise with cooling. The solid formed was allowed to stand for 30 min and filtered off, washed with ether, then with hexane. The product was then isolated as a colourless solid by column chromatography using 10% pet.ether/EtOAc. The single-crystal for X-ray structue anlaysis was obtained from ether solution by slow evaporation.

### Refinement

All the H atoms in (I) were positioned geometrically and refined using a riding model with C—H bond lengths of 0.93 Å and 0.97 Å for aromatic and for methyl  H atoms respectively and *U*_iso_(H) = 1.2*U*_eq_(C) for all carbon bound H atoms.

### Crystal data

**Table:**

C_4_H_5_N_3_O_2_	*F*(000) = 264
*M**_r_* = 127.11	*D*_x_ = 1.436 Mg m^−^^3^
Monoclinic, *P*2_1_/*n*	Mo *K*α radiation, λ = 0.71073 Å
Hall symbol: -P 2yn	Cell parameters from 1804 reflections
*a* = 3.8823 (7) Å	θ = 2.4–25.5°
*b* = 17.499 (3) Å	µ = 0.12 mm^−^^1^
*c* = 8.8171 (17) Å	*T* = 290 K
β = 100.938 (3)°	Block, pale yellow
*V* = 588.12 (19) Å^3^	0.30 × 0.23 × 0.20 mm
*Z* = 4	

### Data collection

**Table:**

Bruker SMART CCD area-detector diffractometer	1098 independent reflections
Radiation source: fine-focus sealed tube	917 reflections with *I* > 2σ(*I*)
graphite	*R*_int_ = 0.016
φ and ω scans	θ_max_ = 25.5°, θ_min_ = 2.3°
Absorption correction: multi-scan (*SADABS*; Bruker, 2004)	*h* = −4→4
*T*_min_ = 0.956, *T*_max_ = 0.977	*k* = −21→20
4285 measured reflections	*l* = −10→10

### Refinement

**Table:**

Refinement on *F*^2^	Primary atom site location: structure-invariant direct methods
Least-squares matrix: full	Secondary atom site location: difference Fourier map
*R*[*F*^2^ > 2σ(*F*^2^)] = 0.039	Hydrogen site location: inferred from neighbouring sites
*wR*(*F*^2^) = 0.116	H atoms treated by a mixture of independent and constrained refinement
*S* = 1.06	*w* = 1/[σ^2^(*F*_o_^2^) + (0.0657*P*)^2^ + 0.085*P*] where *P* = (*F*_o_^2^ + 2*F*_c_^2^)/3
1098 reflections	(Δ/σ)_max_ < 0.001
91 parameters	Δρ_max_ = 0.20 e Å^−^^3^
0 restraints	Δρ_min_ = −0.12 e Å^−^^3^

### Special details

**Table:**

Geometry. All e.s.d.'s (except the e.s.d. in the dihedral angle between two l.s. planes) are estimated using the full covariance matrix. The cell e.s.d.'s are taken into account individually in the estimation of e.s.d.'s in distances, angles and torsion angles; correlations between e.s.d.'s in cell parameters are only used when they are defined by crystal symmetry. An approximate (isotropic) treatment of cell e.s.d.'s is used for estimating e.s.d.'s involving l.s. planes.

### Fractional atomic coordinates and isotropic or equivalent isotropic displacement parameters (Å^2^)

**Table:**

	*x*	*y*	*z*	*U*_iso_*/*U*_eq_	
H3N	0.668 (5)	0.1865 (11)	0.529 (2)	0.069 (6)*	
H1	0.630 (5)	0.3213 (10)	0.510 (2)	0.067 (5)*	
O1	0.2633 (4)	0.39888 (6)	0.89548 (14)	0.0672 (4)	
O2	0.4220 (4)	0.44536 (7)	0.68279 (14)	0.0670 (4)	
C3	0.3702 (4)	0.38941 (9)	0.77686 (18)	0.0502 (4)	
N3	0.5970 (4)	0.21920 (8)	0.59504 (15)	0.0520 (4)	
C2	0.4540 (4)	0.31459 (8)	0.71907 (16)	0.0440 (4)	
N2	0.5029 (4)	0.19156 (8)	0.72315 (16)	0.0577 (4)	
C1	0.5704 (4)	0.29447 (9)	0.58856 (18)	0.0495 (4)	
N1	0.4149 (4)	0.25012 (7)	0.79947 (15)	0.0541 (4)	
C4	0.3391 (8)	0.52244 (11)	0.7273 (3)	0.0937 (8)	
H4A	0.0894	0.5296	0.7056	0.141*	
H4B	0.4472	0.5590	0.6700	0.141*	
H4C	0.4261	0.5295	0.8358	0.141*	

### Atomic displacement parameters (Å^2^)

**Table:**

	*U*^11^	*U*^22^	*U*^33^	*U*^12^	*U*^13^	*U*^23^
O1	0.0984 (10)	0.0524 (7)	0.0599 (8)	−0.0045 (6)	0.0379 (7)	−0.0077 (5)
O2	0.0959 (10)	0.0450 (7)	0.0671 (8)	0.0012 (6)	0.0330 (7)	0.0062 (5)
C3	0.0556 (9)	0.0481 (9)	0.0482 (9)	−0.0045 (7)	0.0137 (7)	−0.0012 (7)
N3	0.0643 (9)	0.0499 (8)	0.0443 (8)	0.0026 (6)	0.0167 (6)	−0.0038 (6)
C2	0.0478 (8)	0.0464 (8)	0.0384 (8)	−0.0034 (6)	0.0095 (6)	0.0015 (6)
N2	0.0754 (10)	0.0480 (8)	0.0532 (8)	0.0009 (6)	0.0213 (7)	0.0017 (6)
C1	0.0597 (10)	0.0499 (9)	0.0410 (9)	−0.0013 (7)	0.0152 (7)	0.0031 (7)
N1	0.0702 (9)	0.0480 (8)	0.0478 (8)	−0.0016 (6)	0.0207 (7)	0.0011 (5)
C4	0.134 (2)	0.0464 (11)	0.1117 (18)	0.0019 (11)	0.0499 (16)	0.0029 (11)

### Geometric parameters (Å, °)

**Table:**

O1—C3	1.2075 (19)	C2—N1	1.3561 (19)
O2—C3	1.3231 (19)	C2—C1	1.360 (2)
O2—C4	1.458 (2)	N2—N1	1.3065 (19)
C3—C2	1.464 (2)	C1—H1	0.906 (19)
N3—C1	1.322 (2)	C4—H4A	0.9600
N3—N2	1.3416 (19)	C4—H4B	0.9600
N3—H3N	0.90 (2)	C4—H4C	0.9600
			
C3—O2—C4	116.63 (15)	N3—C1—C2	104.91 (14)
O1—C3—O2	124.02 (15)	N3—C1—H1	121.4 (12)
O1—C3—C2	124.05 (14)	C2—C1—H1	133.7 (12)
O2—C3—C2	111.92 (13)	N2—N1—C2	108.49 (13)
C1—N3—N2	111.36 (14)	O2—C4—H4A	109.5
C1—N3—H3N	129.6 (12)	O2—C4—H4B	109.5
N2—N3—H3N	119.0 (12)	H4A—C4—H4B	109.5
N1—C2—C1	108.37 (14)	O2—C4—H4C	109.5
N1—C2—C3	120.51 (13)	H4A—C4—H4C	109.5
C1—C2—C3	131.12 (14)	H4B—C4—H4C	109.5
N1—N2—N3	106.88 (13)		
			
C4—O2—C3—O1	−0.7 (3)	N2—N3—C1—C2	0.00 (17)
C4—O2—C3—C2	178.67 (17)	N1—C2—C1—N3	−0.01 (17)
O1—C3—C2—N1	−0.2 (2)	C3—C2—C1—N3	−179.24 (16)
O2—C3—C2—N1	−179.54 (14)	N3—N2—N1—C2	−0.03 (18)
O1—C3—C2—C1	178.95 (17)	C1—C2—N1—N2	0.03 (18)
O2—C3—C2—C1	−0.4 (2)	C3—C2—N1—N2	179.35 (13)
C1—N3—N2—N1	0.02 (18)		

### Hydrogen-bond geometry (Å, °)

**Table:**

*D*—H···*A*	*D*—H	H···*A*	*D*···*A*	*D*—H···*A*
N3—H3N···O1^i^	0.896 (19)	1.980 (19)	2.8659 (19)	169.56 (18)

Symmetry codes: (i) *x*+1/2, −*y*+1/2, *z*−1/2.
